# Ozone high dose therapy (OHT) improves mitochondrial bioenergetics in peripheral blood mononuclear cells

**DOI:** 10.1186/s41231-022-00123-7

**Published:** 2022-07-21

**Authors:** Brigitte König, Johann Lahodny

**Affiliations:** 1 Department of Medical Microbiology and Virology, University Clinic Leipzig, Liebigstrasse 21, Leipzig, Germany; 2 Private Department of Gynecology and General Medicine Univ. Doz. Dr. Johann Lahodny, Klostergasse 1A, St. Pölten, Austria

**Keywords:** Ozone, Major AutoHaemo Therapy (MAH), Mitochondria, Bioenergetics health index (BHI), Peripheral Blood Mononuclear Cells (PBMC), Precision medicine

## Abstract

**Background:**

The worldwide increasing number of people with chronic diseases is pushing conventional therapy to its limits. The so-called Major AutoHaemo Therapy (MAH) has been used in many practices for years. Despite suspicions, especially the 10-passes ozone-high-dosis Therapy (OHT) has shown substantial benefits in chronic ailments. However, knowledge of scientifically based effects of high ozone concentrations are still rare. The present investigation focussed on verifying whether OHT may be linked to a beneficial effect on mitochondrial bioenergetics which can be expressed as a bioenergetic health index (BHI).

**Methods:**

We report on six patients which received OHT for preventive purposes twice within one week. The BHI in peripheral blood mononuclear cells (PBMC) is calculated from parameters of a cellular mitochondrial function assay, which gives insights into different aspects of mitochondrial function: 1) Basal oxygen consumption rate (OCR); 2) ATP-linked OCR and proton leak; 3) Maximal OCR and reserve capacity; 4) Non-mitochondrial OCR.

**Results:**

The results clearly show that the bioenergetic health index in PBMC improves significantly after just 2 OHT applications over a period of 1 week. The overall improvement of the BHI is based primarily on a significant increase in the reserve capacity and the maximum respiration of the mitochondria. The increase in non-mitochondrial oxygen consumption, which has a negative impact on the BHI value, is indicative for the Nrf-2 dependent activation of antioxidant and detoxifying enzymes activated through OHT.

**Conclusion:**

These data demonstrate for the first time the beneficial effect of OHT on mitochondrial parameters. Thus, the results of this study suggest that OHT could be a safe and effective therapeutic option alone or as integrative and complementary support for pharmacological therapy in a variety of chronic and acute diseases where mitochondrial dysfunction plays a central role.

## Background

Worldwide demographic changes and general improvements in healthcare have resulted in a major shift from acute illnesses to chronic, degenerative diseases as causes of human morbidity and mortality. Complex and chronic diseases with underlying mechanisms involving dysfunctional metabolism are a growing healthcare problem in the developed world and enters the countries of the so-called non developed world. Although there are many pharmaceuticals on the market, a cure for advanced chronic diseases is almost not achievable. It is extremely important to look for other therapeutic options which can be used either preventively and / or as an adjunct to a standard therapy. However, these "alternative “ therapies may well be outside of regular conventional medicine at present. Reasons for this are e.g. the fact that promising approaches were not pursued because other forms of therapy were easier to use. Examples include the renaissance of phage therapy in cystic fibrosis due to antibiotic resistant microorganisms [1]. In view of the increasing failure of antibiotic therapies due to multi-resistant microorganisms, the further development of phage therapy is also being considered outside of cystic fibrosis [2, 3]. Another example is the oncolytic virus therapy which is based on reports for more than a century describing the coincidence of various viral or bacterial infections with tumor remission among cancer patients [4].

Ozone therapy has been widely used in everyday clinical practice with increasing use over the last few years and has shown substantial benefits that span a large variety of acute and chronic ailments including herniated discs, cardiovascular diseases, neurological disorders, skin diseases, infectious diseases and pain management [5–10]. However, there is still some need to uncover the physiological mechanism beside its well known antimicrobial and antioxidative capabilities [11, 12]**.** Ozone therapy has many modes of administration. It is often administered by direct intravenous gas (DIV), or by a method called major ozonated autohemotherapy (MAH). MAH was first described in 1954 by Wehrli and Steinbart [13] and since then, after several modification, it has been carried out worldwide millions of times without side-effects and with therapeutic results, albeit poorly documented [14–19]. In brief, Major Autohemotherapy (MAH) is a procedure where around 200 ml blood are mixed with ozone before reinserted under gravity into the patient. For 50 years it was believed that life threatening hemolysis occurs when ozone administration will be repeated within one treatment session. A higher ozone dose and higher ozone concentration method is now known as “L1” 10 passes- ozone high dosis therapy (10 passes OHT) overall in the world. Quite recently, it has been reported in a clinically study that the “L1” 10 passes OHT was a successful addition to conventional therapy [20].

Mitochondria are well known as the power‐generating units of the cell. However, the view of mitochondria acting solely as a powerhouse of the cell is no longer accurate. Besides cell bioenergetics, primary targets of mitochondrial studies include their interplay with essential processes within the cell, including redox and calcium homeostasis, apoptosis and intracellular cell signaling. Thus, it is not surprising that mitochondrial dysregulation or defects have considered as key roles in the aging process and many common disease stages, including, diabetes, atherosclerosis, cancer and neurodegenerative diseases. In 2014 Chacko et al. hypothesized that dysfunctional cellular energetics associated with diabetes, cardiovascular disease, liver disease, cancer and environmental toxins can be dynamically assessed using a new parameter: the Bioenergetic Health Index (BHI) in patient populations [21]. In between it could be shown that this approach can be used as the basis of personalized cell-based measurements to quantify bioenergetic health [22]. The BHI is calculated from parameters of a cellular mitochondrial function assay, which gives insights into different aspects of mitochondrial function. The parameters are mainly 1) Basal oxygen consumption rate (OCR); 2) ATP-linked OCR and proton leak; 3) Maximal OCR and reserve capacity; 4) Non-mitochondrial OCR [23].

A beneficial role of ozone therapy on mitochondrial function could explain the reported positive therapeutic results in various chronic diseases observed specially after the OHT. Thus, we used the BHI as an integrated approach, combining the glycolytic metabolism and oxidative phosphorylation, to generate a cellular bioenergetic profile for peripheral blood mononuclear cells for analysis of their metabolic function during OHT. To our knowledge it is the first study to analyze the effects of OHT on mitochondrial functions.

## Methods

### Patients

In the private department of gynecology and practical medicine of Dr. Lahodny the OHT is used for therapeutic and preventive purposes since 2015. Six consecutive patients who visited Dr. Lahodny for preventive OHT treatments were selected for this pilot study. Routinely, common blood parameters before and during the OHT were taken. During this pilot study the blood which was routinely taken before treatment, immediately after the first treatment, and after the second treatment one week later was used for analysis of mitochondrial functions as well.

### Patient characteristics

The demographic and clinical characteristics of the patients (*n* = 6) enrolled in the study are listed in Table 1.

**Table 1:** Demographic and clinical characteristics of the patients enrolled in the study

Patient	Years	SEX (M/F)	clinic	Actual medication
**1**	**71**	**M**	**diverticulitis in 1991** **cholezystectomy in 2010**	**none**
**2**	**69**	**F**	**No acute/chronic disease**	**none**
**3**	**72**	**F**	**neurodermitis, asthma**	**none**
**4**	**73**	**M**	**No acute/chronic disease**	**none**
**5**	**74**	**F**	**colon cancer in 2012** **chemotherapy in 2013** **no acute/chronic disease**	**none**
**6**	**55**	**F**	**No acute/chronic disease**	**none**

### Ozone high doses therapy (OHT)

In the present case, therapy was admistered by L1 (10-passes) using a Hyper Medozon comfort (Herrmann Apparatebau GmbH, Im Höning 3, 63,820 Eisenfeld, Germany). In this method, 200 mL of patient’s blood is withdrawn from a peripheral vein into a vacuum flask containing of 7500 units of heparin. Oxygen ozone gas mixture, 200 mL, is generated at an ozone concentration of 70 μg/mL The gas is pumped into the bottle under pressure. This pressure is maintained by additional oxygen. Shaking of blood causes a rapid attachment of ozone to plasma and hemoglobin. The blood is then returned rapidly to the patient using positive pressure of 0.9 atmosphere in flask using oxygen. This speeds up the process considerably. This constitutes “one pass” and delivers 14,000 μg of ozone. The procedure was repeated 9 more times with a delivery of 140,000 μg ozone. One L1 (10 passes) lasts approximately one hour.

### Blood collection and cell isolations

While performing the OHT blood samples (1–2 tubes, 8.5 ml/tube) were collected into commercially available cell preparation tubes (CPDA; Sarstedt, Nümbrecht, germany) at the beginning and at the end of the treatment and processed within 24 h of collection. All isolation procedures were designed to prevent activation of the cells during isolation such as performing isolations at room temperature, using RPMI medium (no antibiotics, no phenol red, no FBS) during preparation of leukocytes. Furthermore, OptiPrep™ (STEMCELL Technologies Germany GmbH, Cologne, Germany), which endotoxin levels in each batch are usually measured at < 0.13 EU/ml, was used for the preparation of peripheral blood mononuclear cells (PBMC) according to standard procedures. PBMC were used in a concentration of 2.5 × 10^5^/well.

### Measurement of cellular bioenergetics

Cellular bioenergetics of the isolated PBMC was determined using the extracellular flux analyzer XFe96 (Seahorse Bioscience, Agilent Technologies) and the Seahorse XF Cell Mito Stress Test Kit (Agilent Technologies Germany GmbH & Co. KG, Waldbronn, germany). In this assay the response of cellular oxygen consumption to the sequential addition of mitochondrial inhibitors is used. The final well concentrations (2,5 × 10^5^ PBMC) of oligomycin, FCCP and rotenone/antimycin were 3/3/5 µM. Oligomycin inhibits ATP synthase (complex V), and is injected first in the assay following basal measurements. It impacts or decreases electron flow through the ETC, resulting a reduction in mitochondrial respiration or OCR. This decrease in OCR is linked to cellular ATP production. Carbonyl cyanide-4 (trifluoromethoxy) phenylhydrazone (FCCP) is an uncoupling agent that collapses the proton gradient and disrupts the mitochondrial membrane potential. It is the 2nd injection following Oligomycin. As a result, electron flow through the ETC is uninhibited, and oxygen consumption by complex IV reaches the maximum. The FCCP-stimulated OCR can then be used to calculate spare respiratory capacity, defined as the difference between maximal respiration and basal respiration. Spare respiratory capacity is a measure of the ability of the cell to respond to increased energy demand or under stress. The third injection is a mixture of rotenone, a complex I inhibitor, and antimycin A, a complex III inhibitor. This combination shuts down mitochondrial respiration and enables the calculation of nonmitochondrial respiration driven by processes outside the mitochondria. The experiments were performed in accordance with the manufacturer instructions (Agilent Technologies) and were replicated in six wells and averaged for each experimental condition. The parameter under study were 1) Basal oxygen consumption rate (OCR in pmol/min); 2) ATP-linked OCR and proton leak; 3) maximal OCR and reserve capacity; 4) Non-mitochondrial OCR. Finally, the parameters from the mitochondrial stress test (MST) were integrated as a bioenergetic health index (BHI) [21, 23]. In addition, from these data the cell energy phenotype and the individual metabolic potential can be determined according to the manufacturer`s instructions.

### Statistical analysis

The data reported in the analyses are derived from peripheral blood mononuclear cells (PBMC) isolated from 6 different donors at three different time points. Each PBMC group was comprised of 3–5 technical replicates, and the data is presented as mean ± SEM. Statistical significance was determined using a t-TEST, and p < 0.05 was considered significant. In addition we performed ANOVA for multiple comparisons with an appropriate alpha value and calculated the corrected Cohen's D test as well.

## Results

### Study subjects

Six subjects were enrolled for the analysis of extended mitochondrial function parameters. Their demographic and clinical characteristics are shown in Table 1 in the method section.

### Bioenergetic Health Index (BHI)

**Fig. 1:**
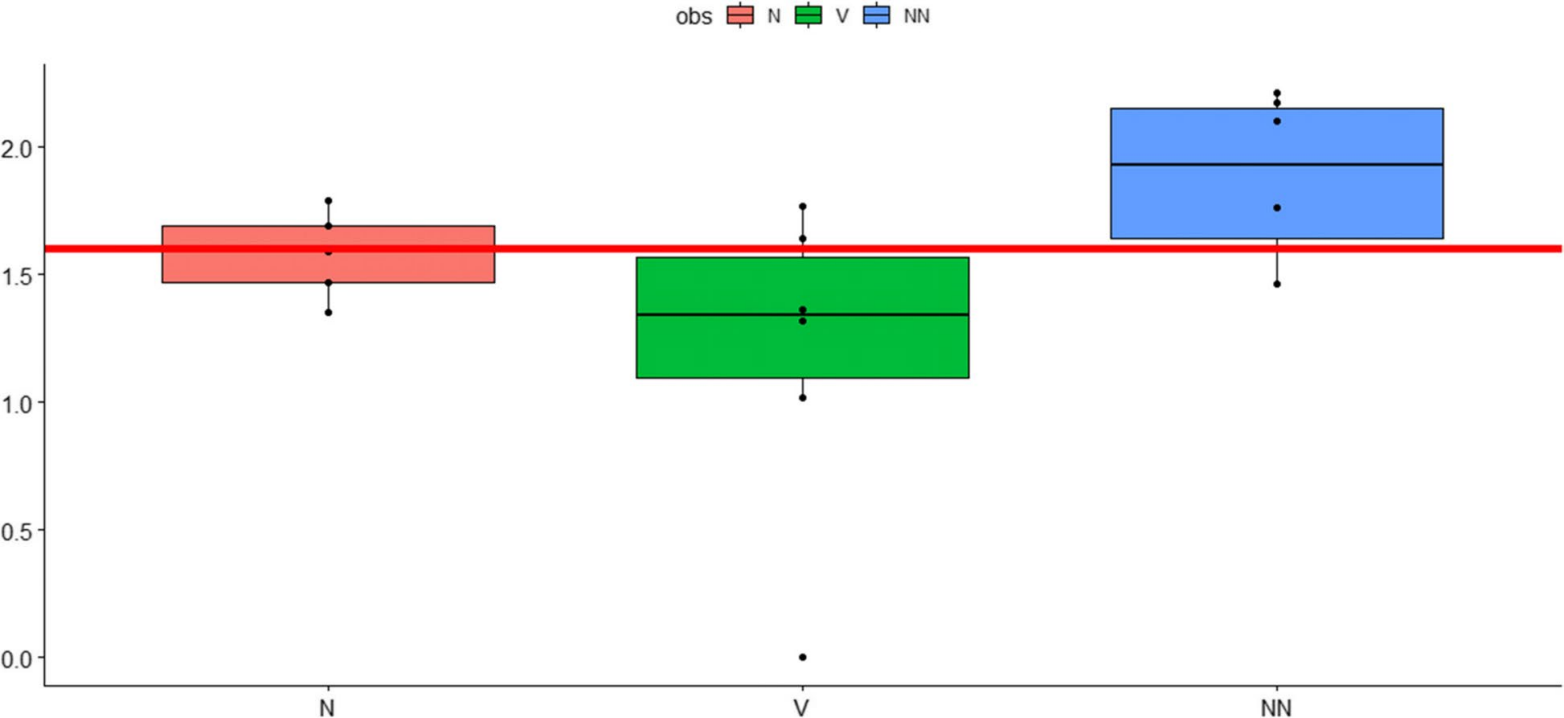
Bioenergetic Health Index (BHI). The individual parameters from the mitochondrial stress test (MST) can be integrated as a bioenergetic health index (BHI). BHI values were determined before, after the first 10-passes OHT and after the second 10-passes OHT one week later. Cumulative data from six patients expressed as mean ± s.e.m. *P*-values: 0,045 (NN versus V); 0,198 (N versus V); 0,079 (N versus NN). COHEN: 1,385,603 (NN versus V); 0,8,039,162 (N versus V); 1,152,394 (N versus NN). ANOVA: 0,0,373,088 (NN versus V); 0,325,814 (N versus V); 0,4,969,881 (N versus NN). V: before OHT; N: after the first OHT; NN: after the 2. OHT (one week later). Y-Axis: BHI; X-axis: sampling time

**Fig. 2:**
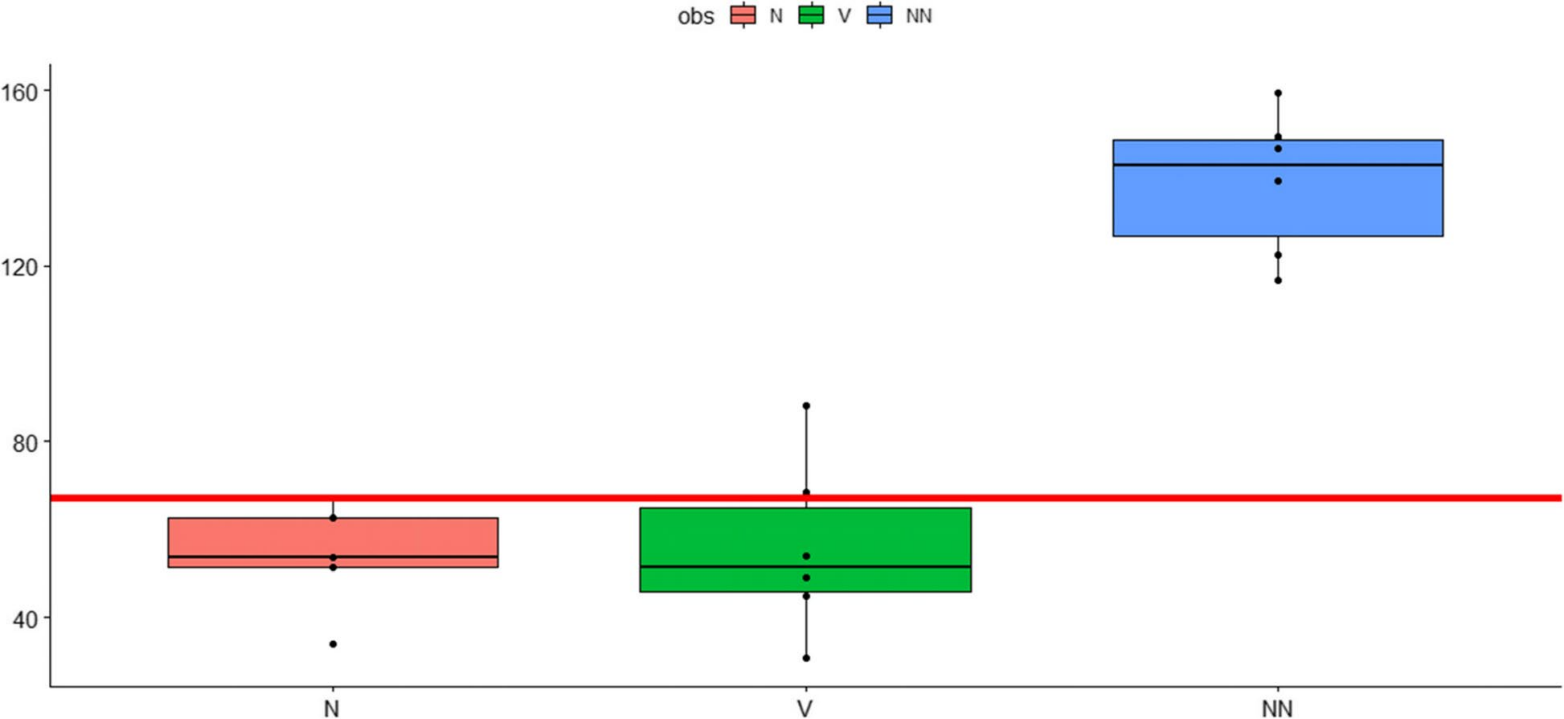
Maximal Oxygen Consumption Rates (OCR). Maximal OCR in pmol O_2_/min were measured before, after the first 10-pass OHT and after the second 10-pass OHT one week later. Cumulative data from six patients expressed as mean ± s.e.m. *P*-values: 0,0,000,172 (NN versus V); 0,88 (N versus V); 0,0,000,471 (N versus NN). COHEN: 4.540905 (NN versus V); 0,1,249,093 (N versus V); 5,725,858 (N versus NN). ANOVA: 0,0,000,019 (NN versus V); 0,9,760,742 (N versus V); 0,00,000,024 (N versus NN). V: before OHT; N: after the first OHT; NN: after the 2. OHT (one week later). Y-Axis: pmol O2/min; X-axis: sampling time

**Fig. 3:**
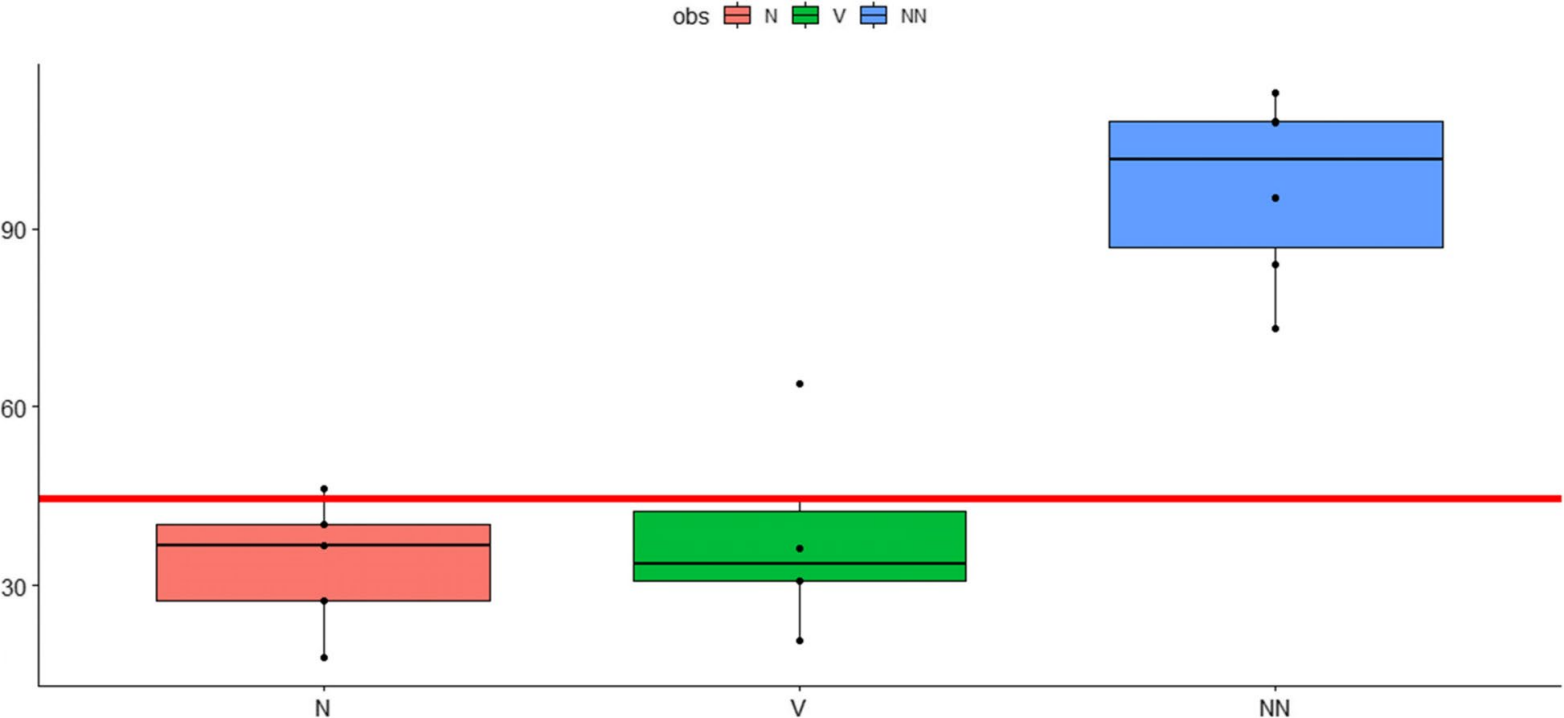
Reserve capacity oxygen consumption rates (OCR). Reserve capacity OCR in pmol O_2_/min were measured before, after the first 10-pass OHT and after the second 10-pass OHT one week later. Cumulative data from six patients expressed as mean ± s.e.m. *P*-values: 0,0,000,567 (NN versus V); 0,614 (N versus V); 0,0,000,308 (N versus NN). COHEN: 3,84,696 (NN versus V); 0,3,071,484 (N versus V); 4,552,083 (N versus NN). ANOVA: 0,0,000,137 (NN versus V); 0,8,829,582 (N versus V); 0,000,011 (N versus NN). V: before OHT; N: after the first OHT; NN: after the 2. OHT (one week later). Y-Axis: pmol O2/min; X-axis: sampling time

**Fig. 4:**
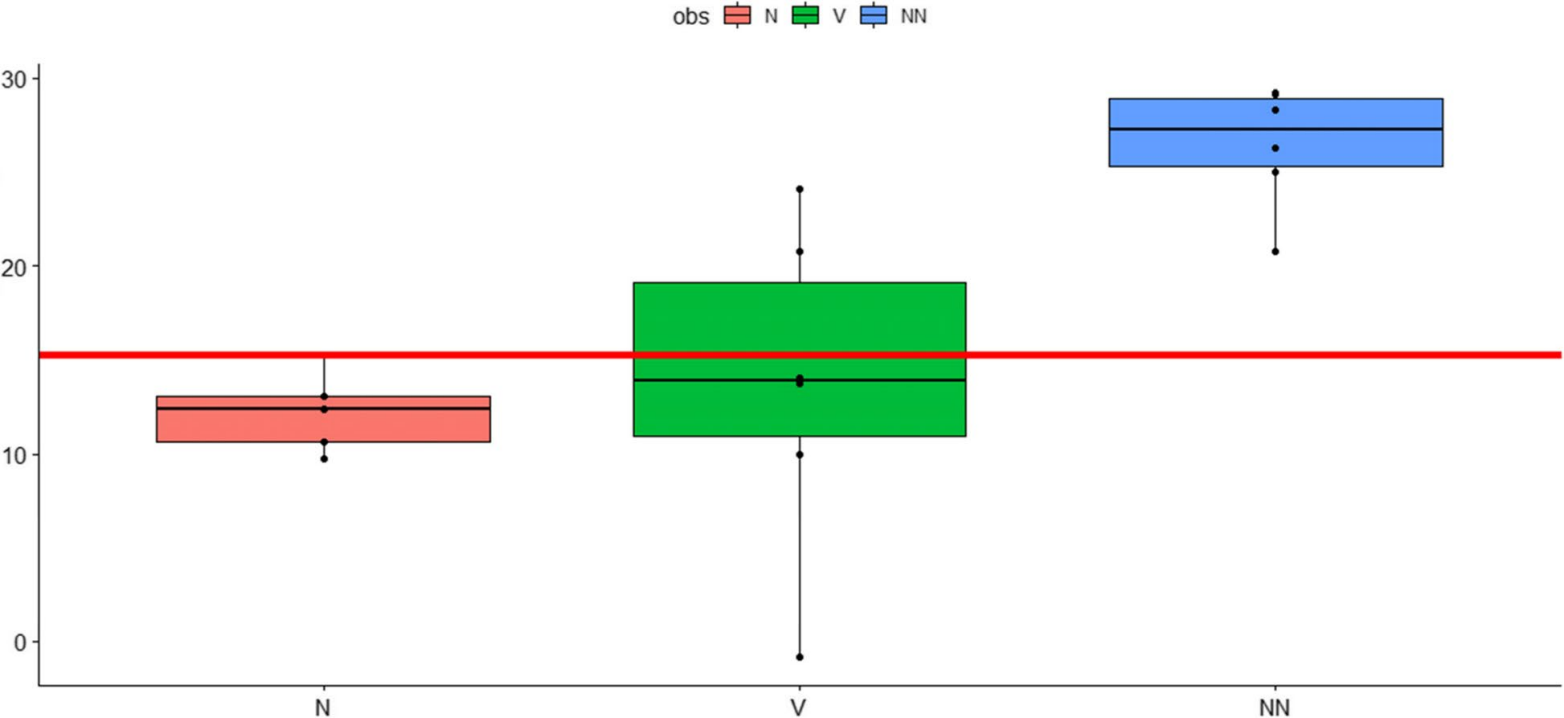
Non-mitochondrial Oxygen Consumption Rates (OCR). Non-mitochondrial OCR in pmolO_2_/min were measured before, after the first 10-pass OHT and after the second 10-pass OHT one week later. Cumulative data from six patients expressed as mean ± s.e.m. *P*-values: 0,01,398,116 (nn versus v); 0,716 (n versus v); 0,0,000,147 (n versus nn). COHEN: 1,939,925 (NN versus V); 0,2,120,872 (N versus V); 5,053,979 (N versus NN). ANOVA: 0,0,043,277 (NN versus V); 0,99,117,745 (N versus V); 0,0,027,815 (N versus NN). V: before OHT; N: after the first OHT; NN: after the 2. OHT (one week later). Y-Axis:pmol O2/min; X-axis: sampling time

We determined the BHI from the 6 patients under study at the three different time points. The cumulative results for the 6 subjects are shown in Fig. 1. The data clearly show that already two sessions of 10-passes OHT increases the BHI significantly. From the mitochondrial stress test (MST) several parameters can be obtained which give insight into specific mitochondrial function. Among the analyzed parameters the maximal oxygen consumption rate (Fig. 2), the reserve capacity (Fig. 3) and the non-mitochondrial oxygen consumption rate (Fig. 4) were significantly affected by OHT treatment. All parameter of the BHI analyzed are summarized in Table 2.

**Table 2:** Parameter of the Bioenergetic Health Index (BHI). The distinct parameter were taken before (v), after the first 10-pass OHT (n) and after the second 10-pass OHT one week later (nn). The data represent cumulative data from the respective subjects and are expressed as means + s.e.m and as median. The statistics include the t-test and the Cohen`t d test

	v (*n* = 6)	n (*n* = 5)	nn (*n* = 6)	overall (*n* = 17)
**Basal respiration** **[pmol O**_**2**_**/min]**				
Mean (SD)	18.0 (5.56)	20.0 (5.30)	42.2 (5.44)	27.1 (12.6)
Median [min, max]	18.0 [9.88, 24.2]	20.9 [13.2, 25.9]	41.2 [36.7, 51.4]	23.9 [9.88, 51.4]
t-test	0.0000182 nn versus v; 0.561 n versus v; 0.0000884 n versus nn			
Cohen`s d	4.39 nn versus v; 4.12 n versus nn			
**Proton leak** **[pmol O**_**2**_**/min]**				
Mean (SD)	1.05 (1.21)	0.874 (0.607)	2.20 (2.42)	1.40 (1.66)
Median [min, max]	0.830 [0.00, 3.27]	1.05 [0.00, 1.42]	1.99 [0.00, 4.83]	0.970 [0.00, 4.83]
T-test	0.33 nn versus v; 0.76 n versus v; 0.24 n versus nn			
Cohen`s d	0.59 nn versus v; 0.71 n versus nn			
**Proton leak [%]**				
Mean (SD)	5.71 (6.59)	4.46 (3.30)	4.76 (5.28)	5.01 (5.03)
Median [min, max]	4.78 [0.00, 17.80]	5.35 [0.00, 7.99]	4.27 [0.00, 11.00]	5.35 [0.00, 17.80]
T-test	0.78 nn versus v; 0.69 n versus v; 0.91 n versus nn			
Cohen`s d	Not applicable			
**Reserve capacity** **[pmol O**_**2**_**/min]**				
Mean (SD)	37.80 (15.00)	33.70 (11.20)	96.90 (15.70)	57.40 (32.90)
Median [min, max]	33.50 [20.60, 63.90]	36.6 [17.8, 46.20]	102 [73.10, 113]	44.60 [17.80, 113]
T-test	0.0000567 nn versus v; 0.61 n versus v; 0.0000308 n versus nn			
Cohen`s d	3.84 nn versus v; 4.55 n versus nn			
**Reserve capacity [%]**				
Mean (SD)	212 (33.30)	179 (84.90)	233 (51.20)	210 (58.80)
Median [min, max]	214 [167, 263]	141 [110, 309]	217 [167, 307]	214 [110, 309]
T-test	0.42 nn versus v; 0.45 n versus v; 0.26 n versus nn			
Cohen`s d	0.47 nn versus v; 0.53 n versus v; 0.78 n versus nn			
**Maximal respiration** **[pmol O**_**2**_**/min]**				
Mean (SD)	55.80 (20.10)	53.70 (12.80)	139 (16.40)	84.60 (44.40)
Median [min, max]	51.40 [30.50, 88.10]	53.6 [33.9, 67.10]	143 [117, 159]	67.10 [30.50, 159]
T-test	0.000017 nn versus v; 0.83 n versus v; 0.0000047 n versus nn			
Cohen`s d	4.54 nn versus v; 5.72 n versus nn			
**Non-mitochondrial respiration** **[pmol O**_**2**_**/min]**				
Mean (SD)	13.60 (8.78)	12.20 (2.16)	26.50 (3.25)	17.70 (8.55)
Median [min, max]	13.90 [-0.88, 24.1]	12.40 [9.72, 15.2]	27.3 [20.8, 29.2]	15.2 [-0.88, 29.20]
T-test	0.0139 nn versus v; 0.71 n versus v; 0.0000147 n versus nn			
Cohen`s d	1.93 nn versus v; 5.05 n versus nn			
**Non-mitochondrial respiration [%]**				
Mean (SD)	39.0 (21.9)	38.60 (9.20)	38.70 (5.14)	38.80 (13.40)
Median [min, max]	48.10 [-5.27, 50.20]	37.60 [29.1, 53.7]	39.8 [32.2, 44.3]	40.6 [-5.27, 53.70]
T-test	0.97 nn versus v; 0.96 n versus v; 0.98 n versus nn			
Cohen`s d	Not applicable			
**Mitochondrial ATP production** **[pmol O**_**2**_**/min]**				
Mean (SD)	17.6 (6.51)	19.20 (5.10)	41.4 (2.94)	26.5 (12.3)
Median [min, max]	16.0 [9.97, 27.6]	19.5 [12.1, 24.5]	40.4 [38.9, 47.0]	23.4 [9.97, 47.0]
T-test	0.0000828 nn versus v; 0.66 n versus v; 0.00011 n versus nn			
Cohen`s d	4.71 nn versus v; 5.49 n versus nn			
**Coupling efficacy [%]**				
Mean (SD)	98.0 (11.1)	96.1 (3.85)	99.2 (9.95)	97.8 (8.63)
Median [min, max]	97.2 [82.2, 115]	94.7 [92.4, 102]	97.4 [89.0, 114]	94.7 [82.2,115]
T-test	0.84 nn versus v; 0.70 n versus v; 0.505 n versus nn			
Cohen`s d	Not applicable			
**BHI**				
Mean (SD)	1.19 (0.637)	1.58 (0.174)	1.88 (0.320)	1.55 (0.508)
Median [min, max]	1.34 [0.00, 1.77]	1.59 [1.35, 1.79]	1.93 [1.46, 2.21]	1.60 [0.00,2.21]
T-test	0.045 nn versus v; 0.19 n versus v; 0.07 n versus nn			
Cohen`s d	1.38 nn versus v; 0.80 n versus v; 1.15 n versus nn			

### Maximal OCR

Basal OCR (oxygen consumption rate) serves as an individual baseline for each subject and varies considerably between individuals. In contrast, the maximal oxygen respiration (after injecting FCCP in the medium) represents a valuable parameter for mitochondrial fitness. A decrease in this parameter is consistent with a deficit in mitochondrial biogenesis, damage to mtDNA or the respiration machinery, or limitations in substrate availability or transport. Figure 2 displays maximal oxygen consumption rates (OCR) in PBMC’s from the 6 patients (cumulative data) before and after OHT. It is clearly evident, that maximal OCR (after addition of FCCP) significantly increased after 2 sessions of 10-passes OHT.

### Reserve capacity

One of the most important parameters of the mitochondrial stress test is the reserve capacity in oxygen consumption rate (pmol/min). The larger the value for reserve capacity the more effectively mitochondria can meet both the ATP needs of the cell and deal with increased energetic demand and ionic or metabolic stress.It is clearly evident from Fig.3 (cumulative data of the 6 subjects) that reserve capacity significantly increased after 2 sessions of 10-passes OHT.

### Non-mitochondrial OCR

The non-mitochondrial OCR represents reactive oxygen species (ROS) generation or other oxygen-consuming processes, including pro-inflammatory enzymes such as the cyclooxygenases and lipoxygenases. However, the activation of anti-oxidative enzymes and enzymes involved in detoxification is reflected by an increase in non-mitochondrial oxygen consumption. It is clearly evident from Fig. 4 (cumulative data of the six subjects) that the oxygen consumption rate is significantly increased after 2 sessions of 10-passes OHT.

### Mitochondrial and glycolytic profiles (Energy Phenotype)

**Fig. 5:**
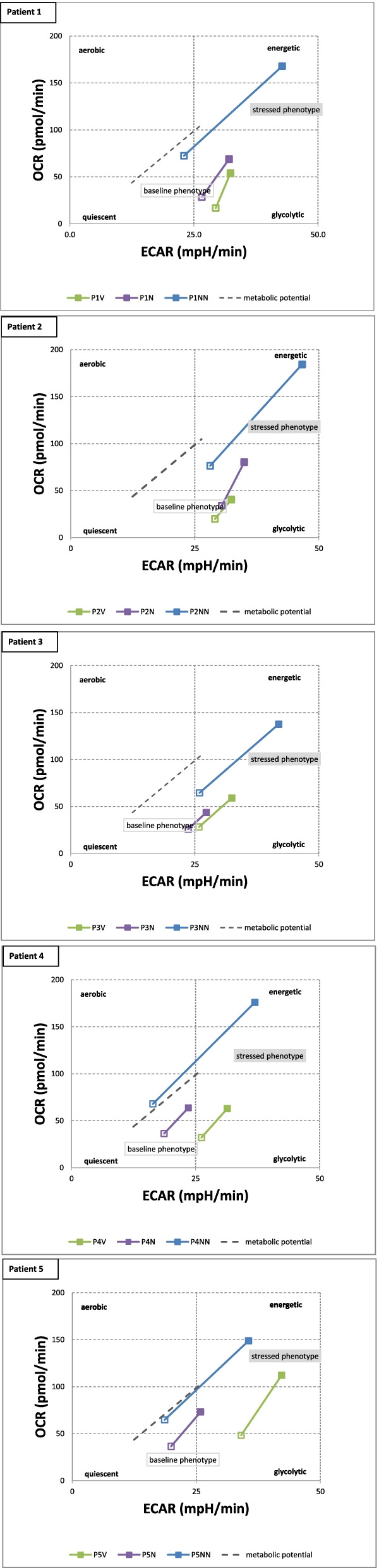
Changes in the mitochondrial and glycolytic profiles (energy phenotype) due to OHT. Metabolic profile of the stimulatory effect of FCCP treatment on aerobic and anaerobic respiration: The OCR and ECAR were plotted against one another at the time where OCR was baseline (baseline phenotype) and where OCR increased to the greatest extent in cells treated with FCCP (stressed phenotype). P1-P5: patients 1 – 5; V: before OHT; N: after the first OHT; NN: after the 2. OHT (one week later). The data are expressed as mean

In addition to the parameters described above we used from the MST profile the following two parameters: a) the extracellular acidification rate in mpH/min (ECAR) and b) the oxygen consumption rate in pmol O2/min (OCR). Both parameters were used to obtain a “metabolic image” of the peripheral blood mononuclear cells (PBMC). The image is divided in four quadrants, which relate the relative activities of the glycolytic and aerobic (mitochondrial) metabolism. As shown in Fig. 5 for each of five patients, OHT stimulates aerobic consumption of oxygen which is indicative of mitochondrial activation. The cumulative data for all analyzed subjects are summarized in Table 3.

**Table 3:** Energy phenotype. The distinct parameter were taken before (v), after the first 10-pass OHT (n) and after the second 10-pass OHT one week later (nn). The data represent cumulative data from the respective subjects and are expressed as means + s.e.m and as median. The statistics include the t-test and the Cohen`t d test. OCR: oxygen comsumption rate in pmolO2/min; ECAR: extracellular acidification rate in mph/min

	v (*n* = 6)	n (*n* = 5)	nn (*n* = 6)	overall (*n* = 17)
**Respiratory quotient, basal**				
Mean (SD)	7.01 (4.04)	5.34 (1.48)	2.09 (0.591)	4.78 (3.23)
Median [min, max]	6.09 [1.65, 13.0]	6.28 [3.63, 6.55]	2.12 [1.20, 2.82]	3.82 [1.20, 13.0]
t-test	0.03 nn versus v; 0.382 n versus v; 0.0055 n versus nn			
Cohen`s d	1.70 nn versus v; 0.52 n versus v; 3.01 n versus nn			
**Respiratory quotient, energy demand**				
Mean (SD)	3.20 (1.21)	2.82 (0.680)	1.48 (0.321)	2.48 (1.10)
Median [min, max]	3.29 [1.50, 5.01]	2.73 [2.22, 3.92]	1.54 [0.95, 1.91]	2.31 [0.95, 5.01]
T-test	0.016 nn versus v; 0.53 n versus v; 0.008 n versus nn			
Cohen`s d	1.94 nn versus v; 2.62 n versus nn			
**Fold increase in OCR,** **energy demand**				
Mean (SD)	2.29 (0.52)	2.04 (0.337)	2.41 (0.211)	2.26 (0.387)
Median [min, max]	2.07 [1.97, 3.32]	2.00 [1.69, 2.42]	2.36 [2.13, 2.71]	2.30 [1.69, 3.32]
T-test	0.622 nn versus v; 0.37 n versus v; 0.077 n versus nn			
Cohen`s d	0.54 n versus v; 1.327 n versus nn			
**Fold increase in ECAR,** **energy demand**				
Mean (SD)	1.29 (0.343)	1.20 (0.0675)	1.87 (0.340)	1.47 (0.411)
Median [min, max]	1.18 [1.08, 1.98]	1.20 [1.13, 1.29]	1.79 [1.53, 2.37]	1.24 [1.08, 2.37]
T-test	0.014 nn versus v; 0.55 n versus v; 0.004 n versus nn			
Cohen`s d	1.69 nn versus v; 2.603 n versus nn			

## Discussion

It is well accepted that mitochondria play a central role in health and disease [24–26]. Using extracellular flux analyses, we evaluated the effects of the OHT on bioenergetic functions in peripheral blood mononuclear cells (PBMC). We show for the first time that OHT improves mitochondrial health [21]. Due to the fact that mitochondrial dysfunctions is at least involved in nearly all chronic diseases OHT might be an useful treatment option in chronic diseases [24, 25]. In addition, mitochondria direct the immune responses to infections as well [26]. Thus, OHT might be an option in acute and chronic infectious diseases as well. We have shown for the first time that ozone therapy might support mitochondrial function and thus increase the bioenergetic health at least in human immune cells.

Up to now the cellular mechanisms accounting for the positive effects of ozonization are not known. In this regard, the effects of low ozone concentrations on cell dynamics and organelles’ structure and function is lacking as well. Mitochondria are known to be very sensitive to even mild oxidative stress and thus they represent an expected target for ozone. In particular, ozone is known to cause alterations of the mitochondrial respiratory chain enzymes [27] and moreover oxidative stress can induce mitochondrial fission [28–30]. The concept of measuring “bioenergetic health” as a diagnostic and/or prognostic clinical tool has gained significant traction over recent years [21–23] However, a major barrier to understanding the role of mitochondria in disease pathogenesis is the complexity of assessing mitochondrial function in humans. In between the measurement of bioenergetics/mitochondrial function in circulating blood cells has emerged as a viable option and represents a minimally invasive method that could be utilized for translational as well as ultimately developed for clinical use in personalized medicine [31–33]. It is now possible, using a mitochondrial stress test, to determine bioenergetic health index (BHI). The BHI is a single value that can define the bioenergetic health in the cells isolated from a patient’s blood. Our data have clearly shown that two L1 (10 passes) treatments led to a significant increase in the bioenergetics health index in human peripheral blood mononuclear cells.

The most important parameters of the mitochondrial stress test are the maximal oxygen consumption rate and the reserve capacity in pmol O2/min. The larger the value for reserve capacity (sometimes-called spare capacity) the more effectively mitochondria can meet both the ATP needs of the cell and deal with increased energetic demand and ionic or metabolic stress. When reserve capacity is zero or negative, the cell cannot satisfy the bioenergetic demand through mitochondrial respiration and the bioenergetic threshold is exceeded [21]. Our data have clearly shown that two L1 (10 passes) treatments led to a significant increase in the maximal consumption rate and the reserve capacity of peripheral blood mononuclear cells (PBMC).

Ozone can be considered a pro-drug, which almost instantaneously reacts with antioxidants and unsaturated fatty acids. These reactions generate the actual ozone messengers represented by either hydrogen peroxide as a fast acting compound or a variety of lipid oxidation products as late effectors. Micromolar amounts of these messengers are able to enhance the delivery of oxygen via erythrocyte activation, the immune system by a bland leukocyte stimulation and most of the remaining body cells by up-regulating the antioxidant system. In this regard, the involvement of the NRF2 protein in vivo was demonstrated. Nrf2’s domain is responsible for activating the transcription of antioxidant response elements (ARE). Upon induction of ARE transcription, an assortment of antioxidant enzymes gains increased concentration levels in response to the transient oxidative stress of O3 [34–36]. The antioxidants induced e.g. superoxide dismutase (SOD), glutathione peroxidase (GPx), glutathione S-transferase (GST), catalase (CAT), heme oxygenase-1 (HO-1), NADPH-quinone-oxidoreductase (NQO-1), heat shock proteins (HSP), and phase II enzymes of drug metabolism. Many of these enzymes act as free radical scavengers clinically relevant to a wide variety of diseases. Nearly all of these mentioned enzymes use oxygen outside the mitochondria. Thus, activation of these enzymes should be correlated with an increase in non-mitochondrial oxygen consumption. Indeed, our data have clearly shown that non-mitochondrial oxygen consumption increased significantly after the second application of OHT. These data extend the previous results on Nrf-2 activation by OHT.

In the past researchers have argued that the total antioxidant status and plasma protein thiol group levels of a blood sample are indicators of the precise amount of O3 required for optimal treatments. By developing more accurate antioxidant status indicators, an individual treatment would achieve the correct dosage on a day and case basis. Some propose to measure simultaneously different biological markers in the blood such as GSH, GPx, GST, SOD, CAT, conjugated dienes, total hydroperoxides, and TBARS. Using an algorithm, information can be gathered about the total antioxidant activity, total pro-oxidant activity, redox index, and grade of oxidative stress. However, we suggest that mitochondrial bioenergetics such as the bioenergetics health index might be the ideal parameter for an individualized ozone therapy.

Ozone therapy stimulates the body toward homeostasis by creating a mild, acute oxidative stress and activates the NRF2 pathway (the same pathway activated during exercise and fasting). Thus, ozone therapy applied in medicine is based on the regenerative capacity of O3 for the treatment of pathological processes such as cardiovascular, peripheral vascular, neurological, degenerative, orthopaedic, gastrointestinal and genitourinary pathologies; as well as multiple sclerosis; fibromyalgia; skin diseases/wound healing; diabetes/ulcers; infectious diseases, lung diseases; osteomyelitis. Quite recently, clinical trials have shown the positive effect of OHT on the outcome of severe COVID-19 disease [20]. However, whether cancer is a contraindication to OZON therapy must be carefully clarified. On the one hand, ozone therapy could support the antioxidant defense of cancer cells. On the other hand, ozone-induced oxygen stress could support the killing of cancer cells. In addition, an optimized immune system is better able to kill the cancer cells [37, 38].

The results presented in the manuscript are deduced from six patients which received OHT for preventive purposes twice within one week. Thus, in further studies more patients should be included. Albeit the documented positive effect of OHT on mitochondrial functions in this paper it is essential that precise guidelines are observed in therapeutic applications and that the concentrations to which the oxygen/ozone mixture is used are within a non-toxic range.

In summary, future studies are now warranted in order to establish a valid confirmation and a more exhaustive explanation underlying the molecular pharmacological and biochemical effects of ozone. Moreover, according to Chacko et. al. an increase in non-mitochondrial oxygen consumption diminishes the BHI value [21]. Thus, the BHI must be assessed individually depending on the physiological cell function.

## Conclusion

The worldwide increasing number of people with chronic diseases is pushing conventional therapy to its limits. The described multi-pass Major AutoHaemo Therapy has been used worldwide in many practices for years quite succesfully. So far, there has been no detailed explanation for the health-promoting properties of OHT. The results of the presented studies have shown that OHT can modulate mitochondrial functions. These studies lay the foundation for the further development of OHT as a safe and reliable method in combating a variety of chronic diseases in an area of precision medicine.

## Data Availability

All data generated or analysed during this study are included in this published article and its supplementary information files.

## Abbreviations

BHI: Bioenergetic health index
MAH: Major AutoHaemo Therapys
OCR: Oxygen consumption rate
OHT: Ozone-high-dosis therapy
PBMC: Peripheral blood mononuclear cells
